# Ventriculo-atrial gradient due to first degree atrio-ventricular block: a case report

**DOI:** 10.1186/1471-2261-5-23

**Published:** 2005-08-09

**Authors:** Giuseppe Ando', Francesco Versaci

**Affiliations:** 1 Cardiac Catheterization Laboratory, Department of Cardiac Surgery, Tor Vergata University of Rome, Italy

## Abstract

**Background:**

Isolated, asymptomatic first degree AV block with narrow QRS has not prognostic significance and is not usually treated with pacemaker implantation. In some cases, yet, loss of AV synchrony because of a marked prolongation of the PR interval may cause important hemodynamic alterations, with subsequent symptoms of heart failure. Indeed, AV synchrony is crucial when atrial systole, the "atrial kick", contributes in a major way to left ventricular filling, as in case of reduced left ventricular compliance because of aging or concomitant structural heart disease.

**Case presentation:**

We performed a trans-septal left atrium catheterization aimed at evaluating the entity of a mitral valve stenosis in a 72-year-old woman with a marked first-degree AV block, a known moderate aortic stenosis and NYHA class III symptoms of functional deterioration. We occurred in a deep alteration in cardiac hemodynamics consisting in an end-diastolic ventriculo-atrial gradient without any evidence of mitral stenosis. The patient had a substantial improvement in echocardiographic parameters and in her symptoms of heart failure after permanent pacemaker implantation with physiological AV delay.

**Conclusion:**

We conclude that if a marked first degree AV block is associated to instrumental signs or symptoms of heart failure, the restoration of an optimal AV synchrony, achieved with dual-chamber pacing, may represent a reasonable therapeutic option leading to a consequent clinical improvement.

## Background

Loss of atrio-ventricular (AV) synchrony due to first-degree AV block may cause important hemodynamic alterations, with subsequent decrease in cardiac output and symptoms of heart failure. Indeed, AV synchrony is crucial when atrial systole contributes in a major way to left ventricular (LV) filling, as in case of reduced LV compliance because of aging or concomitant structural heart disease. We present the case of a patient with first degree AV block, associated with aortic valve disease, who received an evident clinical benefit by sequential pacing despite having refused aortic valve replacement as a first-line therapeutic option.

## Case presentation

**Figure 1:**
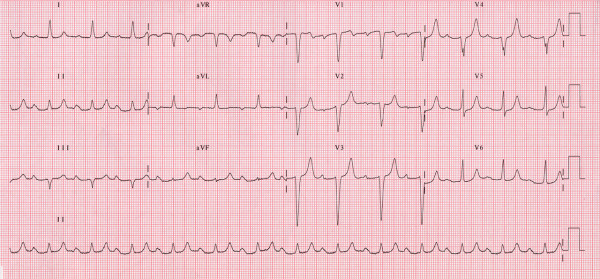
12-lead ECG showing first degree Atrio-Ventricular block. Paper speed is 25 mm/s and scale is 10 mm/mV.

A 72-year-old woman was referred to our Catheterization Laboratory for an invasive re-evaluation of her NYHA class III heart failure symptoms. Three months before she had received, at a different Institution, the echocardiographic diagnosis of moderate aortic and apparently trivial mitral stenosis, despite severe valve calcification, with mild LV hypertrophy (wall thickness was 13 mm) and good systolic function. A pharmacological regimen, consisting in enalapril and furosemide, both 20 mg a day, had been established; despite medical therapy she kept complaining of effort dyspnea. ECG (figure 1) had revealed a first degree AV block, PR interval measuring 290 milliseconds, with normal QRS duration.

**Figure 2:**
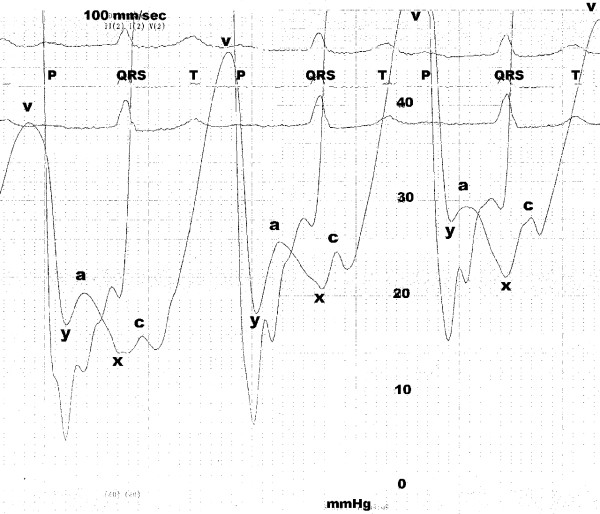
Hemodynamic tracing showing left atrium and ventricle pressures. Capital letters represent ECG waves, whereas small letters represent left atrium waves (see text for details). Scale is 40 mmHg and paper speed is 100 mm/s.

She underwent left and right cardiac catheterization with trans-septal access to the left atrium. Coronary arteries and regional LV systolic function were angiographically normal. LV ejection fraction was 0.65, cardiac index was 2.6 L/min/m^2 ^and aortic and pulmonary systolic pressure were 130 and 38 mmHg, respectively. Aortic stenosis was confirmed to be moderate (the ventricle-to-aorta gradient was 50 mmHg at catheter pullback and aortic valve area was calculated to be 1.0 cm^2^). At fluoroscopy the mitral annulus was heavily calcified, but there was no hemodynamic evidence of mitral stenosis. With the trans-septal technique, moreover, we demonstrated (figure 2) that atrial contraction prematurely occurred soon after mitral valve opening, as shown on the left atrial tracing by the ***a wave ***(corresponding to atrial systole) falling very close to the preceding ***y wave ***(corresponding to early-diastolic unloading of the atrium into the ventricle). The high and prominent ***v wave ***consequently represented marked pressure elevation and reduced compliance of both left atrium and ventricle. Furthermore, LV filling pressure was as increased by the preceding atrial contraction as to cause a 6-mmHg end-diastolic ventriculo-atrial gradient that was evident, at the time of the R wave on the ECG, between the ***x wave ***(corresponding to the atrial relaxation not followed by a properly timed ventricular contraction) and the LV pressure tracing.

We discussed with the patient the therapeutic options [1]: she preferred to defer aortic valve replacement, whereas accepted to undergo permanent pacing. We performed an echocardiographic evaluation of systolic and diastolic function before pacemaker implantation (Table 1). Of note, the functional deterioration could be explained by the evidence of a grade II diastolic dysfunction[2]: transmitral flow was showing a high-velocity (150 cm/s) single filling wave with a deceleration time of 155 ms. Dual-chamber pacemaker implantation with optimal restoration of AV synchrony (programmed AV delay 150 ms) resulted in clinical improvement up to NYHA class II on the same medications. Since both sinus node function and atrial sensing were satisfying, ventricular pacing was triggered by P wave with a QRS duration of 120 ms [3-5]. The echocardiographic evaluation 2 days after pacemaker implantation showed a reduction of both diastolic dysfunction[2] to grade I (pattern of abnormal relaxation with an E/A ratio of 0.6) and pulmonary artery systolic pressure to 28 mmHg, whereas cardiac output appeared to be slightly improved (Table 1) despite the depletive effect of furosemide could have kept the systolic performance lower than expected in such a hypertrophied, non-enlarged left ventricle with aortic stenosis. The patient is now under close echocardiographic monitoring for the optimal timing of aortic valve replacement.

## Conclusion

Isolated, asymptomatic first degree AV block with narrow QRS has not prognostic significance[6] and is not usually treated with pacemaker implantation in clinical practice. Yet, a hemodynamic condition similar to that of pacemaker syndrome [7-10] may occur when a marked first-degree AV block compromises the physiological sequence of events in the cardiac cycle[11]. Indeed, either, with longer PR intervals atrial, contraction takes place during the preceding ventricular systole against closed AV valves[7,10] or, with less long PR intervals, it falls during the early or the mid ventricular diastole, shortening the diastolic period itself and creating an abnormal ventriculo-atrial gradient[11], as in the present case, possibly leading to reversal diastolic AV flow, provided that LV pressure is higher than atrial one during atrial relaxation[9,12,13]. Anyway, the hemodynamic consequences are an increase in pulmonary capillary wedge pressure and a related decrease in cardiac output. Such changes, indeed, are especially harmful both in case of LV systolic[9,14-16] and diastolic (as in the present case) dysfunction and may be, at least in part, antagonized with permanent pacemaker implantation[17,18]. Thus, a properly timed atrial contraction, the "atrial kick", is necessary for optimal LV systolic function by increasing LV end-diastolic pressure while maintaining a low mean left atrial pressure[11].

We conclude that if a marked first degree AV block is associated to instrumental signs or symptoms of heart failure, the restoration of an optimal AV synchrony, achieved by dual-chamber pacing and despite permanent right ventricular stimulation[4,5], may represent a reasonable therapeutic option leading to a consequent clinical improvement.

## Abbreviations

AV: atrio-ventricular

LV: left ventricular

NYHA: New York Heart Association

## Competing interests

The author(s) declare that they have no competing interests.

## Authors' contributions

FV and GA carried out the catheterization procedure. FV coordinated the clinical management of the patient whereas GA prepared, edited and reviewed the manuscript. Both authors read and approved the final draft of the manuscript.

**Table 1:** Echocardiographic parameters in basal conditions and 2 days after pacemaker implantation.

	**Basal**	**2 days after implantation**
LV ED diameter (mm)	41	43
LV ES diameter (mm)	26	26
LV ED volume (ml)	111	123
LV ES volume (ml)	43	43
LV Ejection Fraction (%)	61	65
LV mass (g)	195	197
LVOT VTI (cm)	23	25.5
Stroke volume (ml)	57	64
Cardiac output (L/min)	4.3	4.5
Cardiac index (L/min/m^2^)	2.6	2.7
Pulmonary artery systolic pressure (mmHg)	38	28

LV, left ventricular. ED, end-diastolic. ES, end-systolic. LVOT VTI, velocity-time integral measured at the left ventricular outflow tract. Body surface area is 1,65 m^2^.

## Pre-publication history

The pre-publication history for this paper can be accessed here:


